# Complete Currarino Triad Presenting With Chronic Constipation

**DOI:** 10.7759/cureus.23743

**Published:** 2022-04-01

**Authors:** Habib Y Aldabbab, Hussain A Al Ghadeer, Ashraf A Alnosair, Hussain A Al Jabran, Maram H Alqattan, Chadi M Abdulrahman, Mohammed R Alabbad

**Affiliations:** 1 Orthopedic Surgery, King Faisal University, AlAhsa, SAU; 2 Pediatrics, Maternity and Children Hospital, AlAhsa, SAU; 3 Pediatric Surgery, Almoosa Specialist Hospital, AlAhsa, SAU; 4 General Surgery, Almoosa Specialist Hospital, AlAhsa, SAU; 5 Orthopedics, King Faisal University, AlAhsa, SAU; 6 General Surgery, Armed Forces Hospital, Dhahran, SAU

**Keywords:** sacral defect, anorectal malformation, saudi arabia, chronic constipation, currarino syndrome

## Abstract

Currarino syndrome (CS) is a congenital disorder that is characterized by the triad of anorectal malformation, sacrococcygeal anomalies, and a presacral mass. The inheritance of CS is autosomal dominant. Chronic constipation is the most common symptom of CS. MRI is considered the most sensitive test to diagnose CS. The report describes an eight-month-old baby girl who presented with chronic constipation. Physical examination showed abdominal distension and anal stenosis. Plain radiographs and MRI revealed sacrococcygeal abnormalities with a presacral mass. A patient was diagnosed with Currarino syndrome and managed surgically with excision of the presacral mass and an anorectoplasty via a posterior sagittal midline incision.

## Introduction

Currarino syndrome (CS) is considered a rare congenital disorder. it has been first described in 1981 by a pediatric radiologist, Guido Currarino [1]. CS is characterized by the clinical triad of anorectal malformation, sacrococcygeal abnormalities, and a presacral mass, such as anterior meningocele, being the most common one [2]. The clinical triad of CS is often not complete, and it ranges from mild, to moderate to, severe [3]. The most common clinical presentation of CS is chronic constipation due to external compression by the presacral mass or anorectal malformations [4-5]. We report a case of an eight-month-old infant who was brought to our emergency department for evaluation of chronic constipation and was diagnosed with complete Currarino syndrome.

## Case presentation

An eight-month-old baby girl presented to the emergency department with abdominal distension. She had progressive chronic constipation for the last two months. She had one bowel motion once per week with non-bloody hard stool. It was associated with frequent non-bilious vomiting (food content). Frequent usage of stool softeners and changing formula milk were tried but failed to improve the symptoms. There was no history of engulfing a foreign body. Her parents denied any medical or surgical history for the baby. She passed meconium within the first day of life.

Upon examination, vital signs were within the normal range. Her abdomen was soft, lax, non-tender, moderately distended, and her bowels were distended during palpation. Her back revealed no color changes, abnormal hair growth, or apparent deformity either in the back or lower limbs, which were moving freely with normal muscle tone. Per rectal examination revealed stenotic anus with no gush of stools upon withdrawal of the examiner's finger.

**Figure 1:**
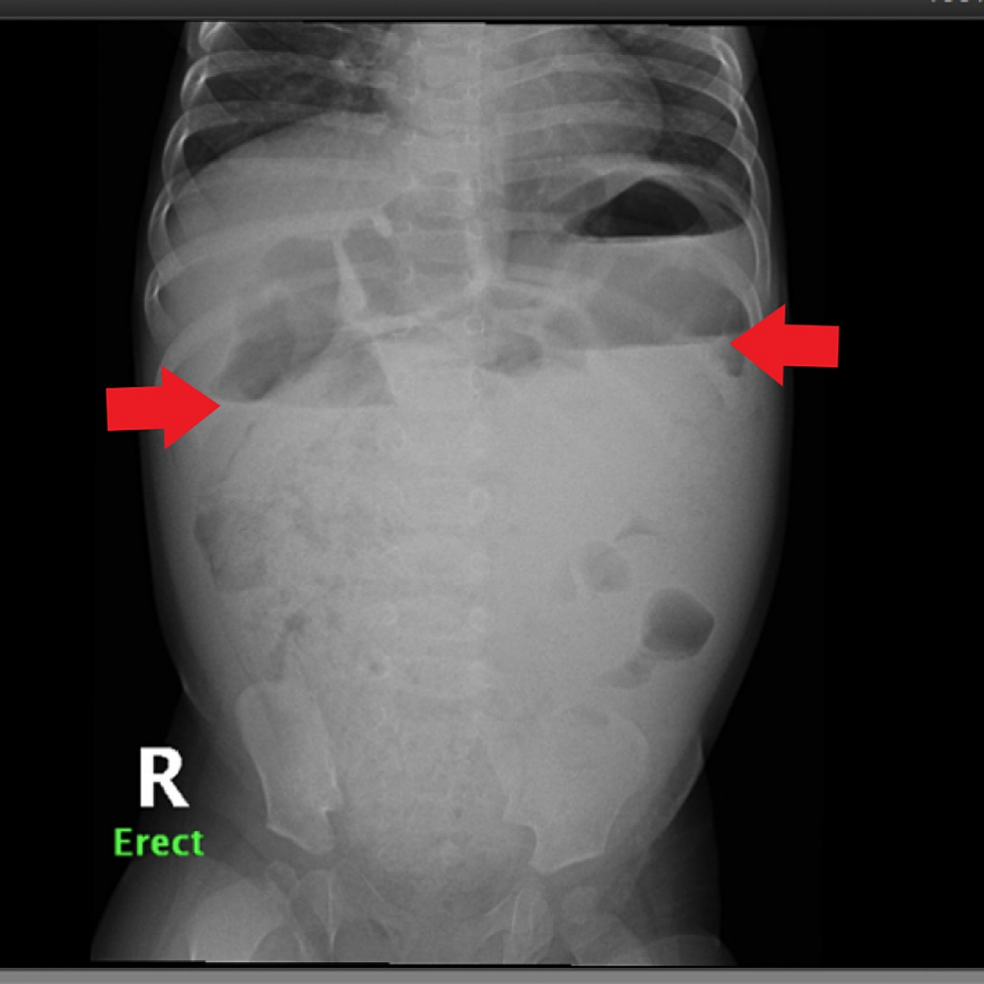
Abdominal erect X-ray showed a distended large colon by air and fecal matter with two air-fluid levels at the ascending and descending parts of the large colon

**Figure 2:**
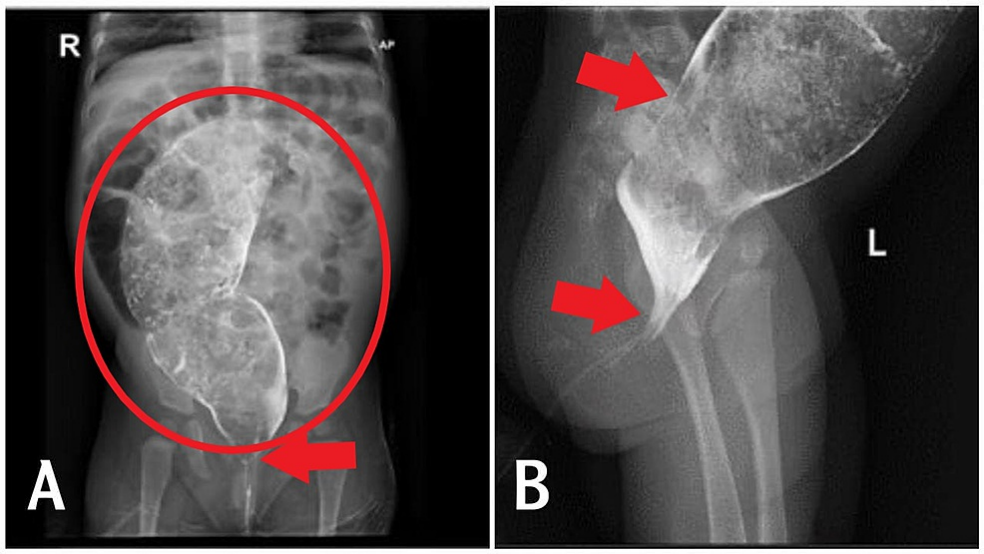
Fluoroscopic barium enema showed evidence of a stenotic short segment at the distal rectum with a significantly distended sigmoid colon

**Figure 3:**
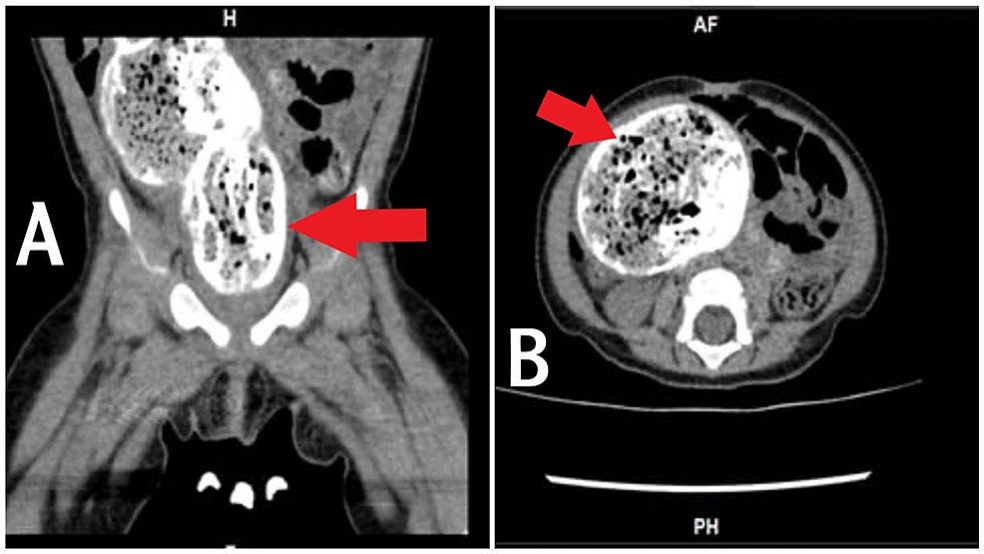
CT of the pelvis without contrast showed megacolon with a high position of the rectal pouch above the level of the puborectalis muscle at the level of S2/S3 A puborectalis muscle cyst is seen with thickened walls measuring around 1×0.5 cm. There is an S5 hemivertebra. The anterior position of the anal canal had irregular, thickened levator ani muscles. There is compression on the bladder by the megacolon. Fecal matter and residual barium contrast are seen.

**Figure 4:**
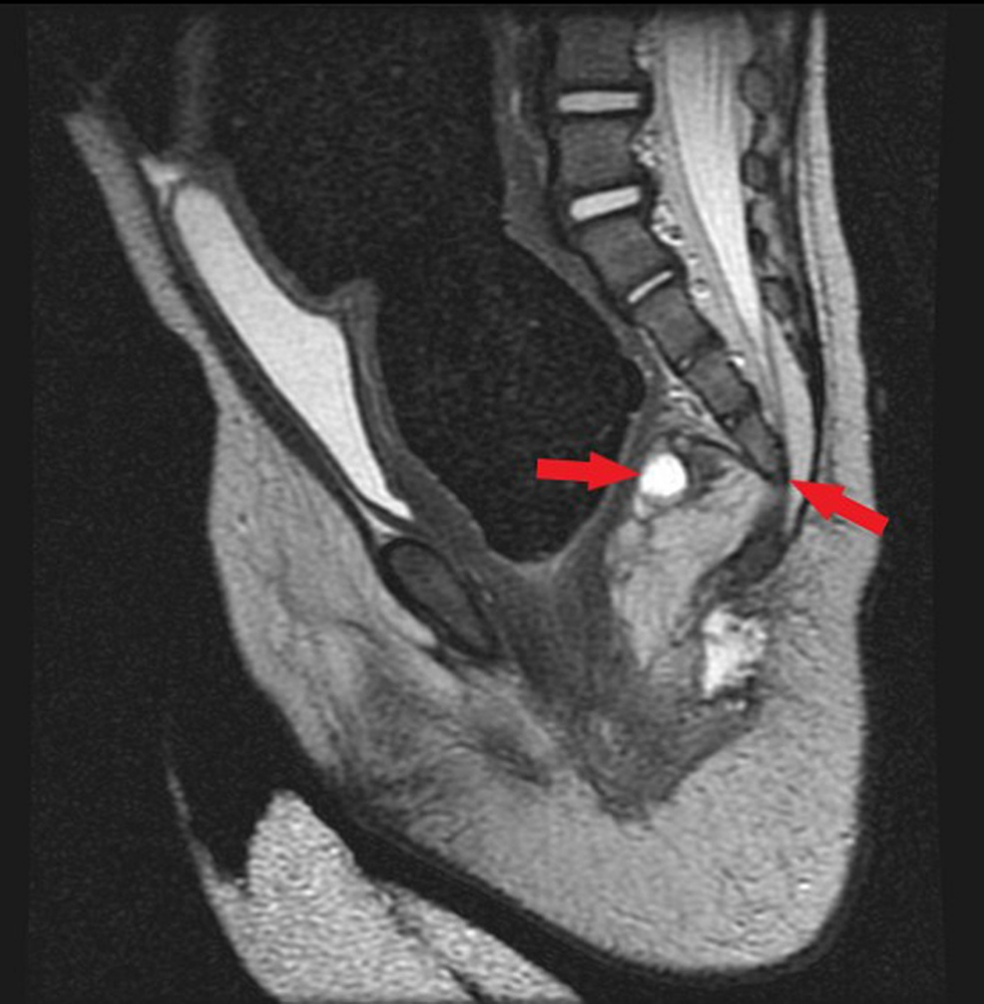
MRI showed there is the redemonstration of a defect at the anterior aspect of the sacrum opposite the S4/S5 level with herniation of a sizable lipoma into the presacral space measuring 2.8 x 2.2 x 3 cm (blue arrow) The distended colon is seen compressing and displacing the urinary bladder anteriorly and superiorly (yellow arrow). A sacrococcygeal osseous defect is detected (red arrow).

PUT/kidney, ureter, and bladder (KUB) X-ray (Figure 1) revealed a distended large colon by air and fecal matter with two air-fluid levels at the ascending and descending parts of the large bowel. A fluoroscopic barium enema (Figures 2A-2B) was done and showed a stenotic short segment at the distal rectum with a significantly distended sigmoid colon. CT pelvis without contrast (Figures 3A-3B) showed a megacolon with a high position of the rectal pouch above the level of puborectalis muscle at the level of S2/S3. A puborectalis muscle cyst is seen with thickened walls measuring around 1×0.5 cm. MRI impressive of presacral lipoma-myelomeningocele (Figure 4). Biopsy of the presacral mass confirmed a mature teratoma. Physical examination and imaging studies suggested Currarino syndrome.

Surgical resection of the mature teratoma was performed via a posterior sagittal approach with anoplasty. The patient suffered from a deep wound infection postoperatively, which was treated with antibiotics and diversion colostomy.

## Discussion

Currarino syndrome has been first described in 1981 by a pediatric radiologist, Guido Currarino [1]. Currarino syndrome is a very rare disorder that occurs because of autosomal dominant inheritance or sporadic [3,6-7]. The pathogenesis behind CS is thought to be a failure of the caudal eminence to be separated from the hindgut and the caudal neural tube leading to the malformations that CS is characterized as, with a triad of anorectal malformation, presacral mass, and sacrococcygeal abnormalities [8]. CS presents as complete or incomplete CS in which only one or two defects are present. Our patient presented with complete CS as anal stenosis, sacral dysgenesis, and a mature teratoma as a presacral mass. A case has been reported as incomplete CS about a 12-day-old patient who presented with necrotizing enterocolitis because of rectal stenosis and presacral mass diagnosed as teratoma, with a normal spine [9].

The most common symptom of CS is chronic constipation due to anorectal malformation, or secondary to a presacral mass, or both as seen in our patient; however, other symptoms have been reported in some cases [5,10]. Presacral masses that have been reported are ventral meningocele, benign teratoma, lipoma, neurofibromatosis, dermoid cyst, enteric cyst, hamartoma, and unclassified tumors [5].

Radiographs and MRI are required to diagnose Currarino syndrome. Radiographs can be used initially to diagnose sacral abnormalities. If the radiographs revealed abnormalities, it should raise possibilities of CS, especially if patients present with unexplained chronic constipation and anorectal malformations as seen in our patient, thus, further imaging studies, such as MRI, are required. MRI is essential for detecting presacral masses and assessment of its extension to the spinal canal. In addition, other anomalies, such as renal anomalies, should be excluded by performing ultrasonography [11-12].

A posterior sagittal midline incision is the preferred approach to explore presacral masses and perform anorectoplasty. A neurosurgeon should be involved and evaluate the presacral mass to minimize nerve injuries that might occur. In the case of teratoma, the coccyx should be removed because of the risk of malignancy, and a follow-up ultrasound and alpha-fetoprotein are essential [5].

## Conclusions

We recommend initial screening with a radiograph for a patient who presents with chronic constipation. If the radiographs raise suspicion of CS, further evaluation with MRI must be done to minimize the complications and decrease the morbidity and mortality that may occur if left untreated.
